# Apical periodontitis after intense bruxism

**DOI:** 10.1186/s12903-022-02123-3

**Published:** 2022-03-24

**Authors:** Madline P. Gund, Karl-Thomas Wrbas, Matthias Hannig, Stefan Rupf

**Affiliations:** 1 Clinic of Operative Dentistry, Periodontology and Preventive Dentistry, Saarland University Hospital, Saarland University, Kirrberger Str. 100, Building 73, 66421 Homburg, Saar Germany; 2 Department of Operative Dentistry and Periodontology, Centre for Dental Medicine, Oral and Maxillofacial Surgery, Medical Centre, University of Freiburg, Freiburg i.Br., Germany; 3 Division of Endodontics, Department of Dentistry, Faculty of Medicine and Dentistry, Danube Private University, 3500 Krems, Austria; 4 Chair of Synoptic Dentistry, Saarland University, Kirrbergerstr. 100, Building 73, 66421, Homburg, Germany

**Keywords:** Apical periodontitis, Bruxism, Occlusal trauma, Cracked tooth syndrome, Perio-endo lesion, Case-report

## Abstract

**Background:**

Bruxism is known to cause masticatory muscle pain, temporomandibular joint pain, headaches, mechanical tooth wear, prosthodontic complications and cracked teeth. Less known to the practitioner, and described only experimentally in literature, is that bruxism can also damage the pulp. To our knowledge, this is the first known clinical case of a patient developing apical periodontitis due to bruxism.

**Case presentation:**

This article presents the case and successful treatment of a 28-year-old healthy male patient with apical periodontitis on teeth 36 and 46 requiring root canal treatment after an intense phase of bruxism. Due to an unclear diagnosis, treatment had been delayed.

**Conclusions:**

Incomprehensible tooth pain can be the result of bruxism. Practitioners need to be informed that intense bruxism can possibly lead to apical periodontitis. It is important, therefore, that a thorough anamnesis is collected and taken into account during diagnostics.

**Supplementary Information:**

The online version contains supplementary material available at 10.1186/s12903-022-02123-3.

## Background

The prevalence of apical periodontitis is as high as 34–61%, it increases with age [1] and is often associated with pulp diseases. A variety of important factors can account for this, including short and long-term irritations (initiated during treatment) or trauma. Trauma can injure a dental pulp by damaging apical blood vessels (from luxation, avulsion injuries, or rupture of intra-pulpal blood vessels), leading to an intra-pulpal haemorrhage. Recovery, repair, or necrosis may follow [2]. The main objective of endodontic treatment is to preserve the tooth in the oral cavity in healthy conditions [3].

Bruxism is described as a nightly orofacial motor function leading to an occlusal trauma, fractures of teeth, headache, muscle pain and periodontal problems. A multifactorial etiopathogenesis is discussed and a mild, moderate and severe bruxism distinguished. Considering neuromuscular activity, a toned, periodic and combined type of bruxism can be classified [4]. In their bibliographic review Demjaha et al. describe a 6–20% prevalence of bruxism in the population in all age groups [4].

Stress, personality characteristics, smoking, disease, trauma, genetics, alcohol, caffeine consumption, illicit drugs and medications may be involved in its aetiology [5]. Typical dental problems caused by bruxism are abrasion, chipping of teeth and/or prosthetic restorations, orofacial pain, teeth sensitivity, pulpal pathology, fractures of teeth and restorations, and damage of implants [6]. As far as the authors know, this is the first described clinical case of apical periodontitis caused purely by bruxism. Experimental consequences of occlusal trauma to the pulp have already been described [7].

## Case presentation

**Fig. 1:**
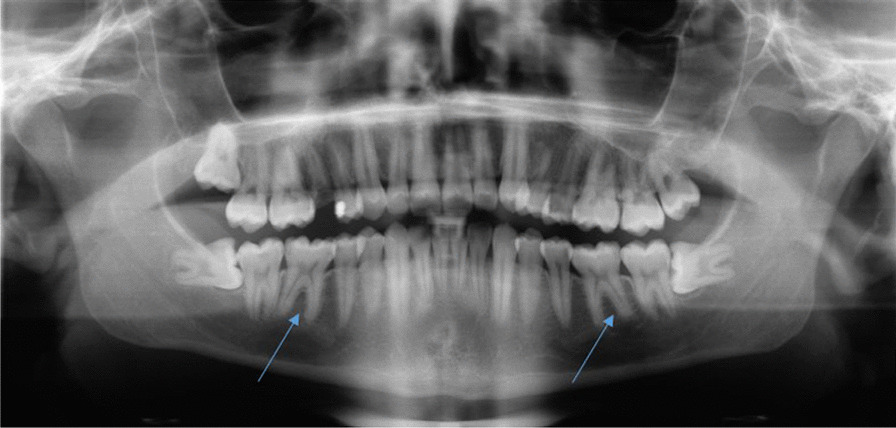
Routine X-Ray taken in January 2019 by the primary dentist. Tooth 36 and 46 without any apical osteolysis before the intensive of bruxism started. Both teeth have no signs of caries and no fillings are visible

**Fig. 2:**
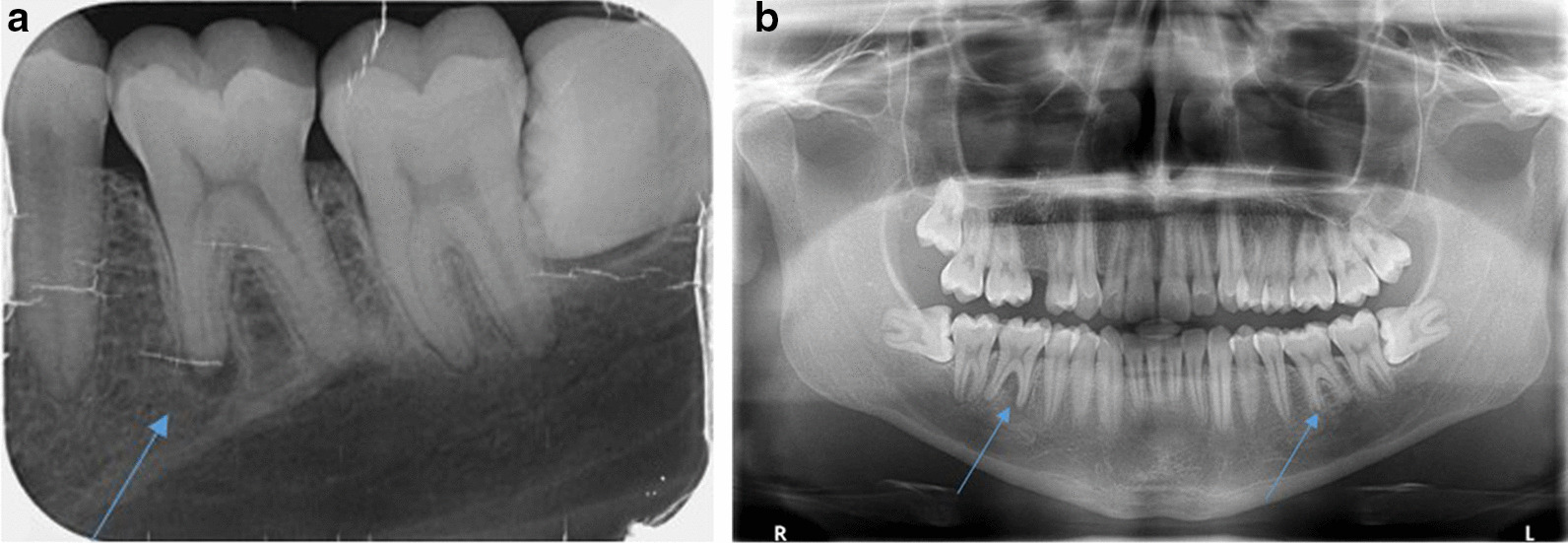
**a** X-Ray taken in October 2019 by the emergency service dentist. Tooth 36 shows an apical osteolysis. No signs of caries and no fillings are visible. **b** X-Ray taken in October 2019 by the emergency service dentist. Tooth 36 and 46 show an apical osteolysis. The apical osteolysis is greater on tooth 46. No signs of caries and no fillings are visible

**Fig. 3:**
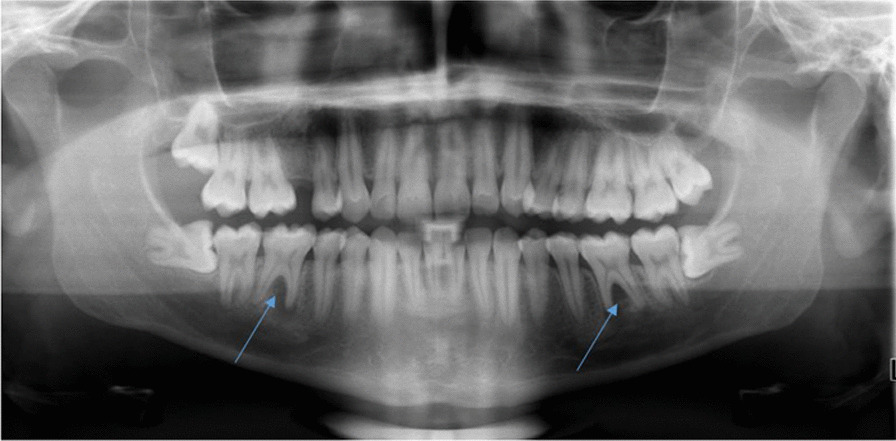
X-Ray taken in January 2020 by the primary dentist. Apical and interradiuclar osteolysis on tooth 36 and 46. No signs of caries and no fillings are visible

**Fig. 4:**
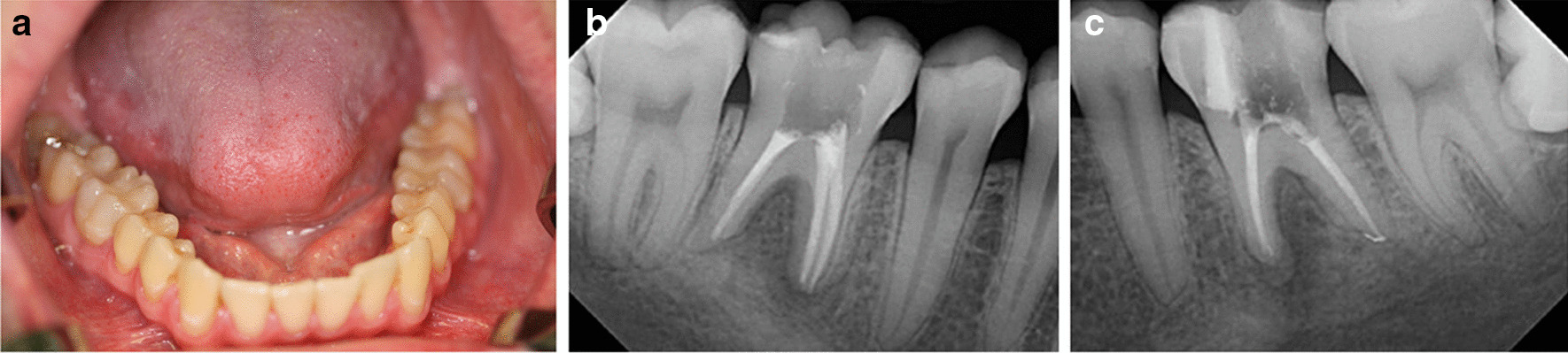
**a**, **b**, **c** Clinical photograph taken in spring 2021. Clear impressions can be seen on each tongue side suggesting that the patient is pressing rather than grinding. Obturation control on tooth 36 and 46 taken in spring 2021. Apical osteolysis on both teeth is clearly declining

A 28-year-old healthy male patient was referred by his primary dentist to the Clinic of Operative Dentistry, Periodontology and Preventive Dentistry, Saarland University in Homburg, Germany with apical periodontitis on teeth 36 and 46. Endodontic treatment had already been initiated, but apical osteolysis was increasing on both teeth. Within the last six months, several changes of medication had taken place. The cause of apical periodontitis could not be determined by the primary dentist. The teeth had no fillings or caries, no trauma or accident had occurred, the patient did not have any periodontal problems, and also had no pain. Clinical examination of both teeth revealed an increased tooth mobility and bifurcation at a probing depth of 4 mm. The periodontal screening index was unremarkable, with no signs of periodontal disease. Teeth 36 and 46 each showed a 10 mm pocket, with bleeding on probing the vestibular-central. Furthermore, a slight bulging elastic swelling could be observed in the vestibulum. The percussion test was negative. Clinically and radiologically no anomalies, pulp calcifications, or formation defects of the hard tooth tissue could be detected. No external resorption, root canal or pulp chamber perforation was apparent. During anamnesis, the patient reported that complaints first appeared 1.5 years ago on the right side (tooth 46). The pain was pulling, diffuse, radiating, difficult to localise and, before going to sleep, pulsating. Simultaneous earache and temporomandibular joint pain occurred. The patient described a short period (2 days) of percussion pain, which then disappeared. The percussion test done by the primary dentist was negative and the sensibility test reduced. The patient did not have any pain on release of pressure. The primary dentist was unable to identify an odontogenic cause for the complaints. Referral to an otolaryngologist prescribing an antibiotic (Amoxicillin 1000 mg, 3 times a day for 7 days) without diagnosis followed, resulting in complete remission of the pain. About 1.5 months later, the same pain started again on both teeth (36 and 46). A short period of percussion pain (2 days) occurred and, once again, disappeared. An emergency service dentist took 2 x-rays (Fig. 2a, b; in comparison: Fig. 1) and gave antibiotics (Clindasaar, 600 mg, 1–1-1), but did not start any treatment, being unable to establish a diagnosis. Remission of pain followed, but 2.5 months later the pain started again. A new x-ray (Fig. 3) was made by the primary dentist, a diagnosis of apical periodontitis on teeth 36 and 46 was finally established, and endodontic treatment was initiated. At that time, the teeth already appeared avital. When explicitly asked by the attending dentist of the university hospital, the patient confirmed having had a lot of stress during the phase of the pain development due to the completion of his studies, as well as having pressed a lot at night (Fig. 4a). For a brief period (1.5 years after the first onslaught of pain and 2 weeks before endodontic treatment), he wore a bite splint. However, his primary dentist could not connect the existing bruxism to the patient’s complaints about teeth 36 and 46. The diagnosis of apical periodontitis indicated by occlusal trauma was established by the attending dentist at the university hospital (for a patient history chart please see Additional file 1). Before the endodontic procedure, and after informed consent was obtained, the patient was requested to rinse his mouth with 12 ml of 0.1% Chlorhexamed Fluid (GlaxoSmithKline, München, Germany). Infiltration anaesthesia was not necessary. The teeth were isolated under a rubber dam (Flexi Dam, Coltene-Whaledent, Alstätten, Switzerland) and the temporary filling removed by ultrasonic device**.** Working length was established using an electronic apex locater (Raypex 6, VDW Dental, München, Germany) and reconfirmed with an intraoral periapical radiograph. The root canals were prepared using Reciproc instruments (VDW Dental, München, Germany) in a torque control endodontic motor (VDW Silver, VDW Dental, München, Germany). During instrumentation, bleeding occurred from the distal canals (teeth 36 and 46) and the patient complained of slight pain. The preparation was carried out with a Reciproc red file (0.25 mm tip diameter, 0,08 taper). Root canal irrigation was carried out with 3% sodium hypochlorite (Hedinger, Stuttgart, Germany), with the rinsing needle placed 1 mm from the working length. Calcium hydroxide powder (Ca(OH)_2_) (Calxyl, Oko Präparate, Dirmstein, Germany) mixed with distilled water was placed as an intracanal medication. The pocket lesions on teeth 36 and 46 were irrigated with 20 ml of 2% of Chlorhexamed Fluid (GlaxoSmithKline, München, Germany). The patient was recalled after 2 weeks for evaluation and did not present with any pain. At the two-week evaluation tooth 36 showed only minimal mobility and tooth 46 none at all. Swelling was no longer detected. The 10 mm pockets on teeth 36 and 46 showed no bleeding on probing. Again, the temporary filling was removed and root canal irrigation with 3% sodium hypochlorite was carried out. During instrumentation, bleeding occurred again from the distal canals, but was reduced (teeth 36 and 46). Calcium hydroxide powder mixed with distilled water was placed once again as an intracanal medication. 4 weeks later, a root canal filling was carried out with gutta-percha (roeko Guttapercha, Coltene-Whaledent, Alstätten, Switzerland; Reciproc Gutta-Percha, VDW Dental, München, Germany) and AH plus (Dentsply Sirona, York, USA) using lateral compaction. After obturation, a radiographic control was performed (Fig. 4b/c), showing a clearly reduced apical osteolysis on both teeth. The access cavity was restored with a nano-hybrid composite restorative material (Herculite, Kerr GmbH, Biberach, Germany). Since then, the patient has remained asymptomatic. The patient has reported no further complaints, and was very satisfied with the treatment.

The next radiographic control will be done 1 year after the root canal filling, and the bite splint will be controlled regularly.

## Discussion and Conclusions

The etiopathogenesis of the apical periodontitis was explained by a diagnosis of exclusion, since neither tooth had caries, fillings, or undergone trauma. No abnormalities in the sense of dental anomalies (Dens invaginatus, Taurodontism), mineralization defects (Amelogenesis/Dentinogenesis imperfecta, Molar-Incisor-Hypomineralisation, Dentin Dysplasia) or (horizontal) tooth fractures could be detected clinically or radiologically. Tooth fractures generally occur from accidents or traumatic injuries and can lead to a periapical lesion. Further radiological examinations are recommended if this is suspected [8]. Since there was no clinical evidence of this and no clues in the patient’s medical history, no further radiological examinations, other than the standard ones, were carried out. At the the beginning of the treatment, a perio-endo lesion can be diagnosed. This is characterized by deep periodontal pockets, a negative or altered pulp response to vitality tests, spontaneous pain and tooth mobility, bone resorption and purulent exudate [9]. These findings apply to the described case as well. In a 2017 classification, the perio-endo lesion was divided into endo-periodontal lesions with and without root damage [10]. Lesions with root damage are divided into external root resorption, root canal or pulp chamber perforation, root fracture or cracking. Lesions without root damage are divided into endo-periodontal lesions in periodontitis patients and non-periodontitis patients [10]. Except for a deep pocket on both teeth, no periodontal problems were detected. The patient, therefore, is considered as a non-periodontitis patient. The deep pockets may emanate from tissues of dental pulp [9]. Since there is no traceable disease of the dental pulp tissue, it cannot be causal. This leaves the possibility of a lesion with root damage. Clinically and radiologically no external resorption, root canal or pulp chamber perforation was apparent. Publications mainly describe vertical tooth fractures in connection with endodontic treatment or root fillings [11, 12]. This can occur due to excessive instrumentation, excessive dentin removal and remaining dentin thickness, excessive irrigation and/or force during lateral condensation. Other causes could include retreatment, overfilled roots, microstructural changes in dentin over a long period, reduced proprioception and fracture resistance of the filled tooth [13]. Since the symptoms associated with a vertical fracture occurred before endodontic treatment, a fracture in the context of treatment was excluded. Several older publications address the vertical fracture of non-endodontically treated teeth [14–16] and describe it as the now widely known cracked tooth syndrome. A cracked tooth is an incomplete fracture of a vital posterior tooth originating from the coronal dentin. Progression in the pulp or periodontal ligament is possible [17]. Vertical fracture of teeth is the third most common reason for tooth loss after caries and periodontitis [18], often associated with intracoronal restorations and mandibular molars [19, 20]. Women are more affected than men, high prevalence rates occur generally in ages 45–64 [19]. There are two different groups of risk factors: (1) iatrogenic (e.g. tooth preparation, width and depth of cavity) and (2) natural factors (tooth form, age, wear patterns) including a lingual inclination of the lingual cusps of mandibular molars, extensive attrition, abrasion, bruxism and clenching. An incomplete tooth fracture is difficult to diagnose and is primarily based on the following symptoms: unexplainable sensitivity to cold, general or localized pain while chewing and pain on release of pressure. Verification involves transillumination with a fiber optic light visualizing the crack, percussion and thermal tests, and radiographs to check the periodontal and pulpal tissue. Ultrasound could visualize future cracks. Currently, there are no existing guidelines for treatment [20]. In this case study, the affected teeth were mandibular molars, but without restorations. The patient was male and under 40. Findings from the National Dental Practice-Based Research Network suggest an incidence rate of 7% in under 35-year-olds [19]. Iatrogenic risk factors can be excluded, as the teeth had never before been treated. The tooth form was not noticeable. The patient did not have an unexplainable sensitivity to cold, only had a short period (2 days) in which he experienced chewing pain, and had no pain on release of pressure. The x-ray showed an impressive apical osteolysis on both teeth. According to the National Dental Practice-Based Research Network, a periapical lucency was detected in 0% of their findings and, therefore, cannot be related to a cracked tooth. Bruxism and stress were reported by the patient and could have been accredited to a cracked tooth [19]. However, since the clinical and radiological picture was not compatible with a cracked tooth, and based on the knowledge that bruxism can damage the pulp [21], this diagnosis was ruled out. Students are under significant stress during their studies and work-life transitions, which can lead, among other symptoms, to bruxism [22]. Over a period of months, the patient reported right temporomandibular joint pain. Since no diagnosis could be established, nonspecific therapy with antibiotics was initiated. Nonspecific therapy with antibiotics should be viewed critically against the background of increasing antibiotic resistance [23]. Furthermore, many side effects of systemic antibiotic administration, including life-threatening side effects, must be taken into account [24]. Therapy for bruxism is extensive: occlusal adjustments, equilibration therapy, occlusal splints, psychotherapy, physical therapy, relaxation training, drugs, biofeedback, and electrical methods [25]. A grinding splint was made 1.5 years after the first onslaught of pain, but no further therapy was given. During this time, apical periodontitis increased. Possibly, an early, adequate therapy for bruxism could have avoided the development of apical periodontitis.

It is likely that the occlusal trauma first led to sterile necrosis, followed by infection. Possible pathways of infection could have been side and accessory canals in the furcation or apical area, both of which are more likely than a cracked tooth, as already discussed.

Treatment was performed according to standard protocol: determination of working length by electrical length measurement using Raypex 6—an established method and product on the market [26], verification of length by radiography—recommended by the European Society of Endodontology [27], irrigation using 3% sodium hypochlorite, which is more effective than the 2% or 1% dose and also considered the most effective irrigation solution in endodontics [28], temporary (2 weeks) drug insertion with CaOH_2_ in case of existing bleeding of distal canals on both molars. The high pH of calcium hydroxide has antibacterial and anti-inflammatory effects, detoxifies bacterial endotoxins and induces healing of the periapical tissues. High healing rates have been reported with the short-term use of Ca(OH)_2_ in teeth with apical periodontitis, and is also an effective antimicrobial agent when applied for a minimum of one week as a temporary filling. Studies suggest an insertion period of 2–4 weeks, when using calcium hydroxide [29]. A new review recommends deciding for or against multiple sessions, depending on the individual case, since no advantages for one particular type of session were found with regard to the incidence and intensity of pain [30]. Obturation was performed using cold lateral condensation which has been, for years, the gold standard. In a 2021 review, cold lateral condensation was compared with warm thermoplastic procedures, both of which failed to achieve complete obturation on micro-CT, but with the thermoplastic procedures achieving better results. However, it was emphasized that the results should be interpreted with caution. Many studies have had a moderate bias. Further studies would be needed to conclusively address the issue [31].

In conclusion, in the case of unexplained tooth pain, possible bruxism should be clarified anamnestically, as this may indicate apical periodontitis in the context of an occlusal trauma. Further case reports and studies are needed to discuss the influence of bruxism on endodontic problems.

## Supplementary Information

**Additional file 1**. Patient history chart: Flow chart of treatment.

## Data Availability

Not applicable.
