# Supplementary material for: Apical periodontitis after intense bruxism
Source: BMC Oral Health. 2022 Mar 24;22:91. doi: 10.1186/s12903-022-02123-3 (PMC8951715; doi:10.1186/s12903-022-02123-3)
Supplement: Supplementary file 1 — Additional file 1. Patient history chart: Flow chart of treatment. [file 12903_2022_2123_MOESM1_ESM.docx]

Patient notices complaints on the right side (tooth 46) 1.5 years ago

with simultaneous earache and temporomandibular joint pain

Patient consults primary dentist.

No diagnosis can be established.

Referral to an otolaryngologist follows.

Otolaryngologist unable to establish a diagnosis.

Prescribes an antibiotic (Amoxicillin 1000mg, 3 times a day,

7 days long) without diagnosis.

Complete remission of pain follows.

1.5 months later: the same pain starts again on both teeth

(teeth 36 and 46).

Emergency service dentist takes 2 x-rays (Image 2a/b).

Gives antibiotics (Clindasaar, 600mg, 1-1-1). Unable to

establish a diagnosis. No treatment is started.

Remission of pain.

2.5 months later pain starts again. New x-ray made by primary dentist (image 3). Diagnosis of apical periodontitis on tooth 36/46 established. Endodontic treatment initiated. Both teeth appear avital. Patient wears bite splint since 2 weeks.

Several changes of medication for half a year. Apical osteolysis increasing on both teeth. Cause of apical periodontitis still unclear. Referral to Clinic of Operative Dentistry, Periodontology and Preventive Dentistry, Saarland University.

The attending dentist at university hospital establishes the diagnosis of apical periodontitis indicated by occlusal trauma. Endodontic treatment on both teeth is completed (image 5a/b).

Additional file 1: Patient history chart.
